# Improving Nurse Participation in Project ECHO: An Institutional Analysis of Primary Health Facilities in Dar es Salaam Region, Tanzania

**DOI:** 10.1002/puh2.70084

**Published:** 2025-08-18

**Authors:** Bahati M. Mfaki, Mackfallen Anasel, Idda Lyatonga Swai, Godfrey Kacholi

**Affiliations:** ^1^ Ministry of Health, Private Health Laboratory Board Dodoma Tanzania; ^2^ Department of Health Systems Management Mzumbe University Morogoro Tanzania; ^3^ Centre of Excellence in Health Monitoring and Evaluation Mzumbe University Morogoro Tanzania; ^4^ Department of Local Governance and Management Studies Mzumbe University Morogoro Tanzania

**Keywords:** ECHO sessions, nurses, participation, Project Extension for Community Health Care Outcomes (Project ECHO), Tanzania

## Abstract

**Background:**

Project Extension for Community Health Care Outcomes (Project ECHO) is recognized as an effective and affordable way to improve healthcare professionals’ knowledge. Project ECHO rapidly expanded in Tanzania from 1 hub to 4 hubs and 12 to nearly 200 spokes in just 3 years. Despite its success, participation among healthcare workers remains low. The study aimed to determine the institutional factors influencing nurses’ participation in Project ECHO sessions.

**Methods:**

This cross‐sectional study was conducted among 450 nurses from 42 selected primary health facilities in Dar es Salaam, Tanzania. A standardized questionnaire was used to collect data from the study participants. We evaluated the association between sociodemographic variables and institutional factors with Project ECHO participation through descriptive analyses and multivariable logistic regression.

**Results:**

Overall, 64% of the study participants had not attended a single ECHO clinic in the past year. The availability of information and communications technology experts to provide technical backstopping to staff (AOR: 0.45, 95% CI: 0.27–0.76, *p* value = 0.003) and the availability of designated rooms equipped with appropriate equipment (AOR: 20.95, 95% CI: 4.44–98.82, *p* value < 0.001) were associated with participation.

**Conclusion:**

The study identifies several factors influencing nurse participation in Project ECHO. These include internet access, incentives, and well‐equipped computer labs, all highly valued. Additionally, the availability of information, communication, and technologies (ICT) experts and effective training significantly contribute to positive experiences. To improve participation, addressing the challenges posed by inflexible scheduling and resource limitations is essential.

## Graphical abstract

Project ECHO has expanded significantly in Tanzania but faces low participation among healthcare workers, particularly nurses. A study of 450 nurses identified key institutional factors affecting attendance, such as the availability of ICT experts and well‐equipped rooms. Addressing scheduling challenges and resource limitations is crucial for improving participation.

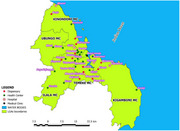

## 1 Background

The Project Extension for Community Health Care Outcomes (Project ECHO) is a videoconferencing community of practice that ensures primary healthcare workers are equipped with knowledge and skills at their workstations [1]. Project ECHO was initiated in 2003 in Albuquerque, New Mexico, to address the disproportionate prevalence of hepatitis C among rural and remote populations. Frustrated by their inability to serve a significant portion of hepatitis C patients due to a shortage of healthcare providers, New Mexico launched this program to mentor community providers statewide in the treatment of the condition [2]. The operation of Project ECHO adopts a hub‐and‐spoke structure through videoconferencing sessions to link specialists (“hub”) at the center with general practitioners (“spokes”) in different regions within the country [1, 2, 3]. The implementation of Project ECHO was successful and effective across the health facilities that used the approach to manage the incidence of hepatitis C, which called for scaling up the approach in other countries to overcome similar health challenges [3].

Project ECHO has been proved in studies to be an effective and cost‐efficient approach for increasing the knowledge of healthcare workers [4, 5]. Both health providers and patients, particularly those in underserved and remote areas, have commended the cost‐effectiveness of Project ECHO across the globe [6, 7, 8, 9]. Results of empirical studies have shown that the use of Project ECHO expanded patients’ access to health care in rural areas while simultaneously improving the skills and practices of healthcare providers, especially those who participated in ECHO sessions [8, 10].

Project ECHO's first program in Africa was launched in Namibia through the HIV ECHO clinic in November 2015 [10]. Project ECHO has grown in more than 13 African countries through the significant support of the Centers for Disease Control and Prevention (CDC), the national public health agency of the United States of America [11]. In Tanzania, Project ECHO was launched in November 2016 and piloted in 12 HIV ECHO clinics (spokes) in three regions from a central location in the city of Dar es Salaam (hub) from November 2017 to September 2018 through the support of CDC under the President's Emergency Fund for AIDS Relief (PEPFAR) [12]. Following the remarkable outcomes demonstrated by Project ECHO, the HIV ECHO clinics were expanded in less than 3 years from one hub and 12 spokes in three regions to four hubs and almost 200 spokes in all 26 regions of Tanzania by 2020 [13].

The Project ECHO received technical assistance from the Center for International Health, Education, and Biosecurity (CIHEB) and the University of Maryland Baltimore (UMB) to expand Project ECHO across Tanzania and set up quality‐control processes [11]. Since 2020, Project ECHO has been coordinated by Mzumbe University through the Centre of Excellence in Health Monitoring and Evaluation (CoEHME). To date, there are 416 spokes and 15 hubs across the country. According to the Project ECHO implementation design, all spokes are required to participate in ECHO sessions from various ECHO clinics, depending on their specific program. Although some sessions, like Adult HIV ECHO clinics, occur weekly, others, such as emerging and re‐emerging diseases, cancer, mental health, and pediatric HIV, meet bi‐weekly. For the purposes of this study, a “clinic” refers to the weekly videoconferencing sessions where healthcare workers of all levels gather to enhance their skills, knowledge, and patient care through ECHO sessions.

Nurses (nurses and midwives combined) are estimated to constitute up to 60% of Tanzania's healthcare workforce and are considered one of the critical cadres required to participate in weekly Project ECHO sessions. Despite the various efforts to ensure that health workers, including nurses, participate in weekly Project ECHO sessions, iECHO data of 2022 show that the overall national participation rate of nurses in the Project ECHO sessions was at 5%. The study seeks to determine the institutional factors influencing the participation of nurses in Project ECHO in primary health facilities in Dar es Salaam Region, Tanzania.

## 2 Materials and Methods

### 2.1 Study Design and Setting

We conducted a cross‐sectional study among nurses working in primary health facilities in Dar es Salaam region. The study was conducted in the five municipal councils of Dar es Salaam: Temeke, Kinondoni, Dar es Salaam, Ilala, Ubungo, and Kigamboni. The region was chosen given that Project ECHO was first implemented in Dar es Salaam before a wider rollout to other regions in Tanzania. Dar es Salaam is the largest and most popular commercial city in Tanzania, with a population of 5,383,72. Ilala (1,649,912), Temeke (1,346,674), and Ubungo (1,086,912) are more populated municipalities, whereas Kinondoni (982,328) and Kigamboni (317,902) are the least populated municipalities. As of 2022, Dar es Salaam had 868 functioning primary healthcare facilities. Of the 868 primary health facilities, 306 (46.4%) were installed with information technology (IT) equipment with Zoom application, two TVs, and a camera to enable healthcare workers to join weekly tele‐mentorship sessions through Project ECHO.

### 2.2 Sample Size and Sampling Procedures

**FIGURE 1:**
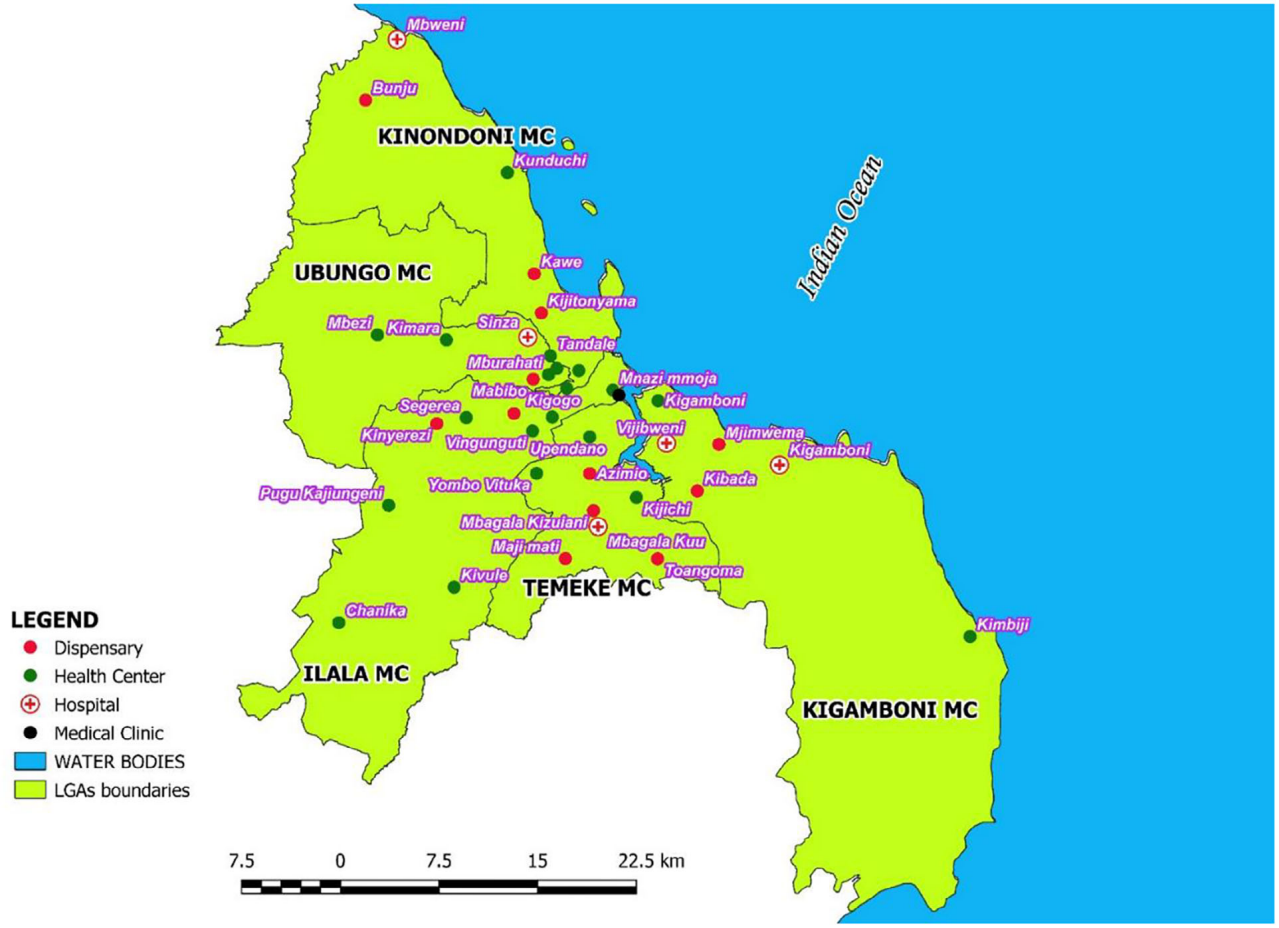
List of facilities that participated in the study (*N* = 42).

The sample size for the study was calculated using Cochran's (1977) formula, which considered a 95% confidence interval and a 5% margin of error. To accommodate a non‐response rate, the sample size was increased by 15%, resulting in a final sample size of 450. The sample size of 450 participants was sufficient for the study to generate the required evidence. We used a multistage cluster sampling procedure to select primary health facilities. The primary health facilities were clustered into three groups: district hospitals, health centers, and dispensaries. From each cluster, 7 district‐level hospitals, 21 health centers, 1 infectious diseases clinic, and 13 dispensaries were randomly selected (Figure 1).

Only primary healthcare facilities with IT equipment and internet bundles from ECHO partners were sampled. These resources were assumed to allow healthcare workers to participate in weekly tele‐mentorship sessions through Project ECHO for at least 2 years before this study. The purposive sampling method was used to select participants from the eligible primary healthcare facilities for the study. The following inclusion criteria were used for selecting study participants: those who consented to take part in the study, those who had at least 12 months of work experience, those who were employed as either full‐time or part‐time, and participants who had heard of Project ECHO before. Study participants were recruited from October to November 2023.

### 2.3 Data Collection Instrument and Procedures

A standardized self‐administered questionnaire was used to collect data from the study participants. To ensure consistency and comparability, only closed‐ended questions were used on the questionnaire. The questionnaire was designed to capture key issues associated with effective participation in Project ECHO sessions. The questionnaire was structured to include [1] socio‐demographic information of participants, [2] participation in Project ECHO sessions, and [3] institutional factors influencing participation in ECHO sessions.

To ensure validity and reliability of the questionnaire, the development of the questionnaire was informed by the recently published literature on related topics [12, 13, 14]; the questionnaire was discussed among the authors (who are also experts in Project ECHO) in order to agree on the nature, scope, and focus of the questions; internal reliability of items used to assess the participation into Project ECHO session was measured using Cronbach's alpha (*α*  =  0.73); and the questionnaire was pre‐tested in six facilities. The results of the pre‐testing were excluded from the overall analysis of this study.

The questionnaire was translated into a commonly spoken national language (Kiswahili) to ensure the participants were able to comprehend it. A well‐trained research assistant who also participated in the pretesting of the data collection tool administered the questionnaires. Interviews were conducted in designated meeting rooms or ECHO equipment rooms within the health facilities after ensuring that no meetings or ECHO sessions were taking place at the time.

### 2.4 Variables and Measurements

Our outcome variable was participation in ECHO sessions. The Project ECHO framework of 2021 defines participation as the act of joining and/or attending Project ECHO sessions to learn, discuss, and share ideas or questions about a particular topic [15]. We accept that ECHO participation in this definition may not be sufficient to cover the broad spectrum of definitions available in the literature; however, such a definition is required for all definition participation and to inform participation studies regardless of which particular definition they use.

Participation in Project ECHO sessions was measured by a Yes or No response to the question, “Have you ever attended ECHO sessions in the last twelve months?” The independent variables were categorized into two: [1] socio‐demographic variables and [2] institutional‐related factors that could affect the participation of nurses in ECHO sessions. The socio‐demographic variables considered relevant to the study included age (measured as a continuous variable), gender (measured as female or male), place of residence (measured as urban or peri‐rural), marital status (measured as married or single), level within nursing cadre (measured as community nurse/medical nurse, enrolled nurse, registered nurse, or other), education level (measured as post‐secondary education or tertiary), working experience (measured as less than 5 years or more than 6 years), and nature of employment (full‐time or part‐time). The institutional factors analyzed in this study were the availability of internet connectivity, power supply, computer labs, qualified information, communication, and technologies (ICT) experts, schedule flexibility, training, and incentives. A binary scale (yes/no) was used to measure each item.

### 2.5 Data Analysis

Data from the questionnaire were retrieved and entered into Microsoft Excel before being exported to STATA version 18 for cleaning and analysis. Descriptive analysis based on frequency, percentages, and measures of central tendency or dispersion (mean, median, or standard deviation and range) was used to understand the situation related to socio‐demographic variables and institutional factors affecting participation in ECHO sessions. We used logistic regression analysis to determine the association between the outcome variable (participation in ECHO sessions) and independent variables (socio‐demographic and institutional factors), and a *p* value of less than 0.05 was considered significant. Independent variables statistically significant in univariate analyses were considered for inclusion in multivariable models.

## 3 Results

### 3.1 Characteristics of the Participants

We obtained data from 450 participants, 354 (78.7%) of whom were more than 26 years of age, and the median age was 34 years (IQR: 27–44). Most study participants were female (79.8%, *n* = 359) and resided in urban areas (79.6%, *n* = 358). A significant portion of the sample identified as registered nurses (68.9%, *n* = 310). More than 70% (*n* = 347) had more than 6 years of work experience in their respective healthcare facilities. The majority (96.2%, *n* = 433) were full‐time staff. Table 1 provides details on participant characteristics (Table 2).

**TABLE 1:** Character of the study participants (*n* = 450).

Variable	Frequency	Percentage (%)
Age group		
≤ 25 years	96	21.3
≥26 years	354	78.7
Median age	34 (IQR: 27–44)	
Gender		
Male	91	20.2
Female	359	79.8
Place of residents		
Rural	52	11.6
Urban	358	79.6
Peri‐urban	40	8.8
Marital status		
Single	266	59.1
Married	184	40.9
Profession		
Community nurse/Medical	54	12.0
Enrolled nurses (EN)	68	15.1
Registered nurse (RN)	310	68.9
Nursing administrators	18	4.0
Education background		
Tertiary (college/university)	450	100
Work experience at the health facility		
≤5 years	103	22.9
≥6 years	347	77.1
Nature of the work		
Full time	433	96.2
Part‐time	17	3.8

**TABLE 2:** Status of participation in Project Extension for Community Health Care Outcomes (Project ECHO) sessions.

Items	Frequency	Percentage (%)
Ever attended the ECHO clinic in the past 12 months in at least one session		
No	288	64
Yes	162	36
Frequency of attendance into ECHO sessions		
1–3 months	71	43.8
4–6 months	86	53.1
7–9 months	5	3.1
Types of ECHO sessions with participants frequently participated in		
Adults HIV clinic	46	28.4
Pediatric HIV clinic	71	43.8
TB clinic	23	14.2
Blood safety	11	6.8
Covid‐19	11	6.8
Number of ECHO sessions attended in the past 12 months		
1–10	103	63.6
11–20	43	26.5
21–30	11	6.8
31–40	5	3.1
41 and above	0	0

### 3.2 Status of Participation in Project ECHO Sessions

The study revealed that 64% (*n* = 288) of healthcare workers had not attended a single ECHO clinic in the past year. Most attendees (53.1%, *n* = 86) participated in 4–6 monthly sessions, indicating consistent engagement. Pediatric HIV clinic was the most frequently attended session type, accounting for 43.8% of participant involvement. The HIV adults, TB, and COVID‐19 clinics had significant participation rates, ranging from 6.8% to 28.4%. More than half (63.6%) of attendees participated in 1–10 sessions, whereas a smaller proportion (36.4%) attended 11 or more sessions.

### 3.3 Facility‐Related Factors That Affect the Participation of Nurses in Project ECHO Sessions

The strongest factors identified to promote nurse participation in Project ECHO sessions were the availability of internet facilities (95.1%; *n* = 428), the availability of incentives to motivate nurses’ participation in ECHO sessions (91.3%; *n* = 411), and the availability of computer laboratories with conducive working spaces (90%; *n* = 405). The availability of ICT experts to provide technical support, as well as adequate internally organized ICT training, was perceived to positively influence nurses’ participation in Project ECHO sessions (Table 3). Two main factors that were perceived to negatively affect nurse participation in Project ECHO sessions were the inflexibility of the schedule for ECHO sessions (82%; *n* = 369) and the unavailability of alternative power sources in the health facilities (78%; *n* = 351) (Table 3).

**TABLE 3:** Facility‐related factors that affect participation in Project Extension for Community Health Care Outcomes (Project ECHO) sessions (*n* = 450).

Items	Response	
	Yes *N* (%)	No *N* (%)
Availability of internet at the facility	428 (95.1)	22 (4.9)
Availability of alternative source of power at the facility	99 (22.0)	351 (78.0)
Availability of computer laboratory with conducive working spaces	405 (90.0)	45 (10.0)
Availability of ICT experts to provide technical backstopping	345 (76.7)	105 (23.3)
Flexibility of ECHO sessions’ schedule	81 (18.0)	369 (82.0)
Availability of training on information and communication technologies	345 (76.7)	105 (23.3)
Availability of incentives to motivate nurses’ participation in ECHO session	411 (91.3)	39 (8.7)

### 3.4 Association Between Facility‐Related Factors and Participation of Nurses in the Project ECHO Sessions

The logistic regression analysis shows that there was an association between participation and availability of ICT experts to provide technical backstopping when needed (AOR: 0.45, 95% CI: 0.27–0.76, *p* value = 0.003) and availability of a computer laboratory with conducive working spaces (AOR: 20.95, 95% CI: 4.44–98.82, *p* value < 0.001). No statistical association was found between participation and health facilities with internet accessibility, alternative power sources, and flexible ECHO sessions’ schedules (Table 4).

**TABLE 4:** Association between facility‐related factors and participation of nurses in the Project Extension for Community Health Care Outcomes (Project ECHO) sessions (*n* = 450).

Items	Ever attend ECHO the past 12 months		COR, 95% CI	*p* value	AOR, 95% CI	*p* value
	Yes *N* (%)	No *N* (%)				
Availability of internet at the facility						
No	7 (31.8)	15 (68.2)	1		1	
Yes	155 (36.2)	273 (63.8)	1.22, 0.49–3.05	0.676	0.43, 0.13–1.49	0.184
Availability of alternative source of power at the facility						
No	132 (37.6)	219 (62.4)	1		1	
Yes	30 (30.3)	69 (69.7)	0.72, 0.45–1.17	0.182	0.65, 0.37–1.17	0.149
Availability of computer laboratory with conducive working spaces						
No	4 (8.9)	41 (91.1)	1		1	
Yes	158 (39.0)	247 (61.0)	6.56, 2.30–18.66	<0.001	20.95, 4.44–98.82	<0.001
Availability of ICT experts to provide technical backstopping						
No	44 (41.9)	61 (58.1)	1		1	
Yes	118 (34.2)	227 (65.8)	0.72, 0.46–1.13	0.151	0.45, 0.27–0.76	0.003
Flexibility of ECHO sessions’ schedule						
No	137 (37.1)	232 (62.9)	1		1	
Yes	25 (30.9)	56 (69.1)	0.76, 0.45–1.27	0.289	0.72, 0.39–1.29	0.268
Availability of training on information and communication technologies						
No	38 (36.2)	67 (63.8)	1		1	
Yes	124 (35.9)	221 (64.1)	0.99, 0.63–1.56	0.963	0.71, 0.41–1.22	0.218
Availability of incentives to motivate nurses’ participation in ECHO session						
No	12 (30.8)	27 (69.2)	1		1	
Yes	150 (36.5)	261 (63.5)	1.29, 0.64–2.63	0.477	0.63, 0.21–1.85	0.399

## 4 Discussion

The purpose of this study was to determine the institutional factors influencing the participation of nurses in Project ECHO in primary health facilities in Dar es Salaam Region, Tanzania. This study was carried out as part of a larger study that examined the factors that influence nurses’ participation in ECHO sessions in Tanzania.

The findings of the current study suggest that participation of nurses into Project ECHO sessions was low. Low participation in ECHO sessions could be attributed to the scheduling and frequency of the ECHO sessions, which may not be convenient for all participants [16]. Previous studies have shown that nurses, predictably, have challenging schedules that may involve shift rotations, prolonged hours of work, and a wide range of responsibilities within and outside health facilities [17, 18, 19]. Consequently, scheduling ECHO sessions that accommodate their accessibility and availability is essential. Although the ECHO sessions last 60 min a week, nurses could find it difficult to find time to participate due to a shortage of health staff and patient influx in their facilities. Although the current study did not assess engagement and interactivity, previous studies have demonstrated that low engagement and interactivity may dissuade participation [20].

This study identified technical issues such as stable internet connectivity, bandwidth, and the availability of ICT professionals for technical support as potential facilitators of ECHO session participation. Most participants in the facilities (spokes) were able to connect with a team of specialists (hubs) via Zoom videoconferencing at scheduled times. This could be attributed to the Zoom platform's user‐friendliness, which is easier and more versatile to use and does not require sophisticated ICT‐related technological skills [21]. In addition, the study suggests that nurses with internet access could access learning materials such as online courses, research articles, and multimedia materials, which are often shared during ECHO sessions. However, it is important to note that this finding contradicts the findings of a study conducted by resource‐poor countries during epidemic situations that identified internet connectivity as a barrier to the practical and appropriate utilization of telemedicine [22]. The variation in results could be attributable to differences in healthcare environments, geographical locations, or infrastructure availability among areas.

Participants in our study, like those in prior studies, acknowledged that the availability of incentives was one of the factors that influenced their decision to participate in the ECHO sessions [20]. This is in part due to the architecture of ECHO program, which provides opportunity for health professionals to earn CPD points for each session they attend and use for relicensure. Additionally, studies have demonstrated that healthcare workers are motivated to participate in ECHO sessions to enhance their knowledge and confidence [20, 23]. Learning new skills and competencies for handling patients who would otherwise be referred to the next level of care could also be one of the main motivations for participating in ECHO sessions [24]. Similarly, by participating in ECHO sessions, nurses can boost professional satisfaction while decreasing isolation.

Our findings demonstrated that the availability of alternative sources of electricity at the healthcare facilities was an important factor influencing nurses’ participation in ECHO sessions. This could be associated with the fact that at least 78.4% of Tanzania's mainland total population had access to electricity in 2020. However, this finding contradicts the findings of a retrospective program evaluation that examined ECHO participants’ performance in the management of HIV/AIDS patients in 10 Zambian provinces and identified power outages as a barrier to ECHO implementation [21]. It is especially important to note that participation in ECHO sessions often requires access to devices such as computers or tablets. These devices rely on electricity to function along with being connected to the internet. Without electricity, nurses would be unable to participate in virtual sessions.

Many nurses participated in the ECHO sessions because specific computer rooms with favorable environments were established for the ECHO activities in the surveyed facilities. In Tanzania, all facilities (spokes and hubs) have designated rooms equipped with appropriate equipment such as video‐conferencing systems, cameras, monitors, and other tools to ensure optimal visual and audio quality for successful interaction during ECHO sessions [15]. The designated rooms not only encouraged nurses to participate in the sessions but also provided a consistent and standardized atmosphere for ECHO sessions, enabling at least all participants, regardless of location, to have a similar experience.

### 4.1 Strengths and Limitations

This study has the following strengths: First, to the best of the author's knowledge, this is the first study to be undertaken in Tanzania. Second, participants were drawn from three primary levels of health service delivery (district hospitals, health centers, and dispensaries). However, given the study was entirely quantitative and conducted in Dar es Salaam (Tanzania's largest city and financial hub), the generalization of the results may be limited.

## 5 Conclusion

The level of participation into ECHO sessions among nurses was low. The availability of ICT experts to provide technical backstopping and internet connectivity, as well as designated rooms for ECHO activities, was the key factor that influenced nurses’ participation in ECHO sessions. A rigid schedule for ECHO sessions was a significant obstacle for nurse participation, with 82% of respondents identifying it as a challenge. To improve nurse involvement in Project ECHO sessions, it's essential to implement more flexible schedules that cater to the varying needs and constraints of healthcare professionals. At the same time, it's important to ensure that a pool of ICT experts is readily available to provide timely technical support. Additionally, regularly assessing participation rates will help in developing strategies to boost attendance in ECHO sessions.

## Author Contributions

Bahati M. Mfaki conceptualized, designed the study, and collected the study. Godfrey Kacholi analyzed and interpreted the data and drafted the manuscript. Mackfallen Anasel and Idda Lyatonga Swai reviewed the manuscript. All authors approved the final version of the manuscript.

## Ethics Statement

This study adhered to rigorous ethical standards to ensure the protection of participants’ rights and well‐being. Prior to participation, all study participants were fully informed about the study's objectives, potential benefits, risks, and the voluntary nature of their involvement. Participants were clearly informed that their participation was entirely voluntary and that they had the right to withdraw from the study at any time, without any obligation or need to provide a reason.

## Consent

Written informed consent was obtained from each participant, ensuring that they understood the purpose, procedures, and their rights throughout the research process.

## IRB Statement

This study underwent a comprehensive ethical review process and received formal approval from the Directorate of Research and Postgraduate Studies at Mzumbe University (reference number MU/DPGS/INT/38/Vol.IV/284). Ethical clearance was also granted by the National Institute for Medical Research (NIMR) under reference number NIMR/HQ/R.8a/Vol.IX/4435. Additionally, permission to access study participants was obtained from the leadership of Dar es Salaam Region, as well as from relevant authorities within the health facilities where the research was conducted. These stakeholders acted as gatekeepers, facilitating access to the study population.

## Conflicts of Interest

The authors declare no conflicts of interest.

## Data Availability

Please contact the authors regarding the availability of the raw data in text format only. For reasons of confidentiality, raw data will not be made available.
